# Treatment Intricacies in Mandibular Implant-Supported Rehabilitation of a Patient With Down Syndrome: A Clinical Report

**DOI:** 10.7759/cureus.31148

**Published:** 2022-11-06

**Authors:** Hariharan Ramakrishnan, Surabhi Halder, Mirza Rustum Baig

**Affiliations:** 1 Department of Prosthodontics and Implantology, Ragas Dental College and Hospital, Chennai, IND; 2 Department of Restorative Sciences, Kuwait University, Jabriya, KWT

**Keywords:** implant-supported overdentures, edentulism, implant supported removable prosthesis, microstomia, down's syndrome

## Abstract

Edentulism is considered a poor health condition and may compromise the quality of life. Prosthodontic replacement of missing teeth results in significant improvement of oral functions. Treating a patient with Down syndrome can pose clinical challenges in handling the emotional aspect as well as in rendering treatment. Careful oral analysis and diagnosis of the existing conditions in such patients will enable and pave the way for clinically acceptable treatment results. This clinical report describes the challenges encountered in the prosthodontic management of an edentulous young patient with Down syndrome.

## Introduction

Complete dentures have been a standard treatment of choice for edentulous patients. This treatment strategy restores standard functionality and esthetics in edentulous patients [1]. These treated individuals have raised multiple complaints of difficulties of adaptation, most of which have been associated with mandibular complete dentures [2,3]. Today, implant-supported overdenture remains a good option for these patients. Residual ridge resorption can interfere with the success of mandibular complete denture retention. Restoration of an edentulous mandible with dual implants‑supported overdenture is a well-accepted treatment with successful long-term outcomes. It is considered the standard of care [4]. Van Steenberghe et al. were among the first authors to propose two implants in the edentulous mandible in 1987 [5]. McGill's consensus statement on overdentures said that a two-implant-retained overdenture should be the minimum treatment for the edentulous mandible [6]. The advantages include good facial support in the presence of advanced mandibular resorption, the need for only two implants to reduce treatment costs, good occlusal stability for the opposing prosthesis, and easy removal of the prostheses for facilitating oral hygiene procedures [3,5].

Prosthesis fabricated over dental implants provides increased retention, stability, and support compared to conventional dentures and improves masticatory functions and facial esthetics. Hybrid dentures are fixed complete dentures in the mouth using dental implants and overcome the disadvantages of removable dentures, which require frequent removal and cleaning. They consist of acrylic resin teeth and denture bases attached to a metal framework connecting the implants. This is known as FP3-fixed prosthesis type 3. Unlike conventional dentures, the flanges do not extend up to the vestibular depth but stop at the attached gingiva region and are more comfortable for the patient [4].

Hybrid dentures provide very good retention and resist the dislodging forces directed toward the alveolar bone. Fixed prosthesis provides psychological stability and significantly improves masticatory ability. It has a stimulatory effect that delays resorption. Esthetics, taste perception, and phonetics are also better when clinically compared with conventional complete dentures since the palatal surface is not covered [3,6].

Down syndrome, trisomy 21, is a genetic autosomal and chromosomal alteration due to additional genetic material from abnormal cellular division. 14.47 in 10,000 live births occur with this anomaly. Multiple common medical and dentofacial manifestations are reported in these patients [7,8]. The standard dentofacial features include tooth macrostructure anomalies like hypodontia, malocclusion, dentition wear due to bruxism, decreased occlusal vertical dimension, chronic periodontal disease, hypotonic orofacial musculature, decreased salivary flow pattern, high occurrence of dental caries. An underdeveloped maxillary arch and mandibular prognathism are common bony defects in these patients [9]. These dentofacial manifestations can cause alteration of the masticatory apparatus [10]. Intellectual disability varies widely and may affect their overall behavior during dental procedures. Dental management mostly depends on the clinical level of this disability [11]. In addition to the oral manifestations, compromised cooperation may add to the complexity of the treatment. Therefore, the bond of trust between the patient and dentist is significant for better treatment outcomes. The type of treatment depends on the age, intellectual disability, severity of the oral manifestations, and the operator's skills and knowledge [12].

This clinical report presents the practical challenges in rehabilitating a young female patient with Down syndrome and a one-year follow-up.

## Case presentation

A 23-year-old female patient was referred to the Department of Prosthodontics from the Department of Oral Surgery and the Department of Periodontics with a chief complaint of missing teeth in the lower arch. The patient expressed a desire for a denture. The patient had no relevant past medical history. The patient was emotional by nature. On extra oral examination, the patient presented with short stature, short and curved extremities, bowed legs, and frontal bossing with bilateral facial symmetry. There was partial microstomia and ectodermal dysplasia. The patient's parents and sister had a history of short stature. Intraoral examination revealed that the patient had previously visited the nearby hospital and had undergone extraction of several malformed upper and lower teeth without any postoperative complaints. The patient already had a fixed implant-supported hybrid prosthesis in the maxilla and four implants in the mandible placed two years back in the Surgery and Periodontics department of the same institution.

Other clinical findings included Achondroplasia. It is a gene aberration with an autosomal dominant pattern of inheritance whose essential clinical feature is dwarfism caused by a gene mutation in the fibroblast growth factor receptor 3 (FGFR3) gene [7,9,11].

**Figure 1:**
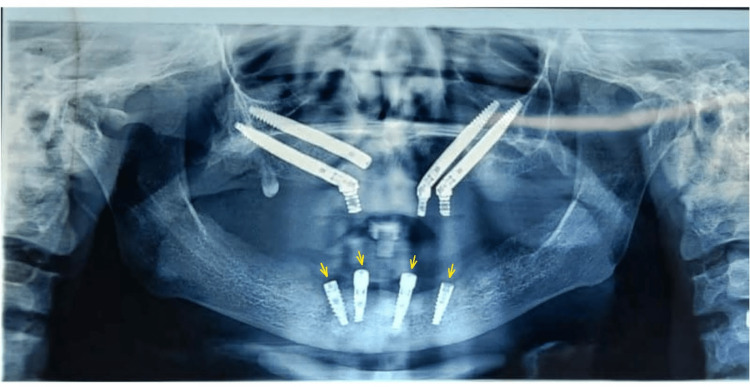
Pre-Prosthetic Orthopantogram showing four angulated implants in the mandibular anterior region (arrows)

Two mesial implants in 42,32 regions (FDI Notation, Norris Medical, Israel) and two angled distal implants in 34,44 (FDI Notation, Norris Medical, Israel) regions were seen during the intraoral examination of the mandible (Figure 1). 

Treatment options and the cost involved in restoring the mandible were explained in detail and discussed with the patient, and the patient opted for an implant-supported hybrid prosthesis in the mandible. The patient desired to report after four months for prosthodontic treatment.

**Figure 2:**
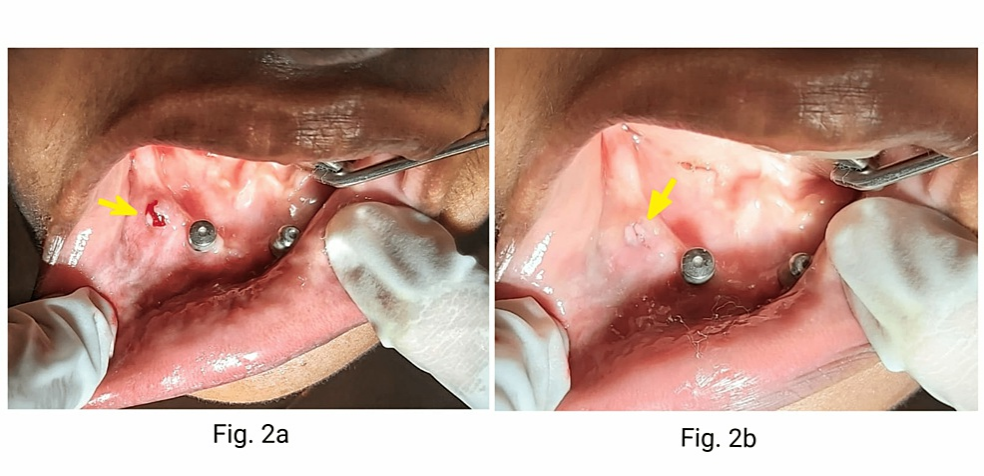
2a: Epulis-like growth subsequently incised (arrow), 2b: The healed area is seen after one week( arrow )

**Figure 3:**
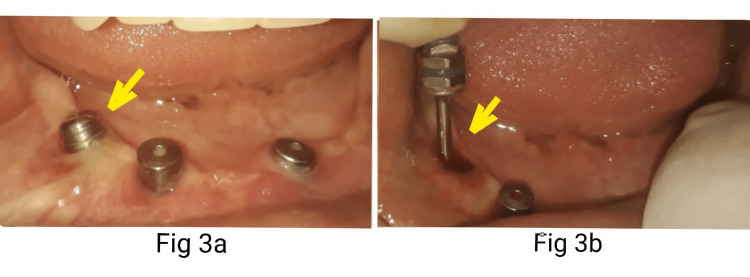
3a: Right distal implant submerged using a gingival collar (arrow), 3b: created by healing abutment (arrow).

After four months, when the patient returned to the department, she changed her mind about the treatment plan. She opted for an implant-supported overdenture instead of a hybrid prosthesis which she had accepted previously. Therefore, it was decided to convert the distal implants into fully submerged dummy implants and utilize the mesial implants for two implant-supported overdenture. The right distal implant with a cover screw was already well closed by soft tissue, but clinically, there was a soft tissue bulge representative of small epulis-like tissue growth on the right distal implant due to a loosened cover screw. Under LA, the small epulis-like structure was carefully removed (Figure 2a, 2b) and the cover screw was tightened. Complete clinical soft tissue closure of this implant was done by intentionally placing healing abutment (height 2mm,dia 2mm, Norris Medical, Israel) to achieve soft-tissue collar height and utilize the same for closure and submerging of the implant (Figure 3a, 3b).

**Figure 4:**
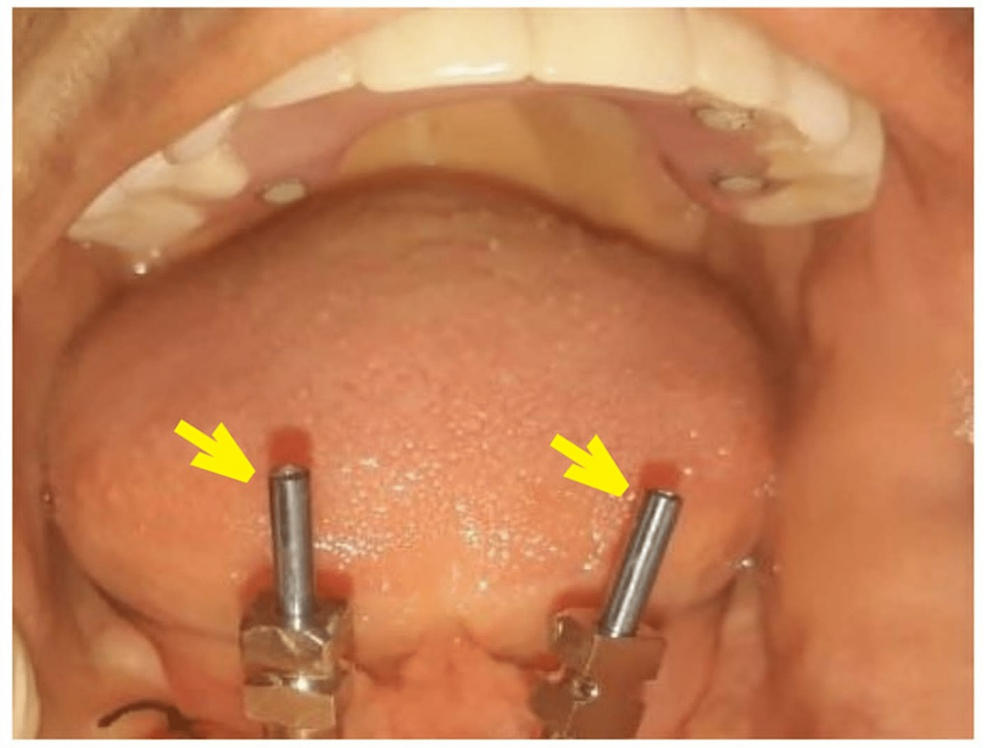
Divergent open tray impression copings of mesial implants (arrows).

Under LA, the mesial implants were exposed, and healing abutments were placed. (height 3mm, Norris Medical, Israel). Two weeks later, an excellent gingival collar was achieved in 32, 42 regions (FDI Notation), and there was complete healing of the right distal implant soft tissue previously involved with growth. Due to partial microstomia, the length of open tray impression copings (H12, SS, Norris Medical, Israel) was close to the upper hybrid denture. Therefore, the height was ground and reduced to 10 mm (Figure 4). 

**Figure 5:**
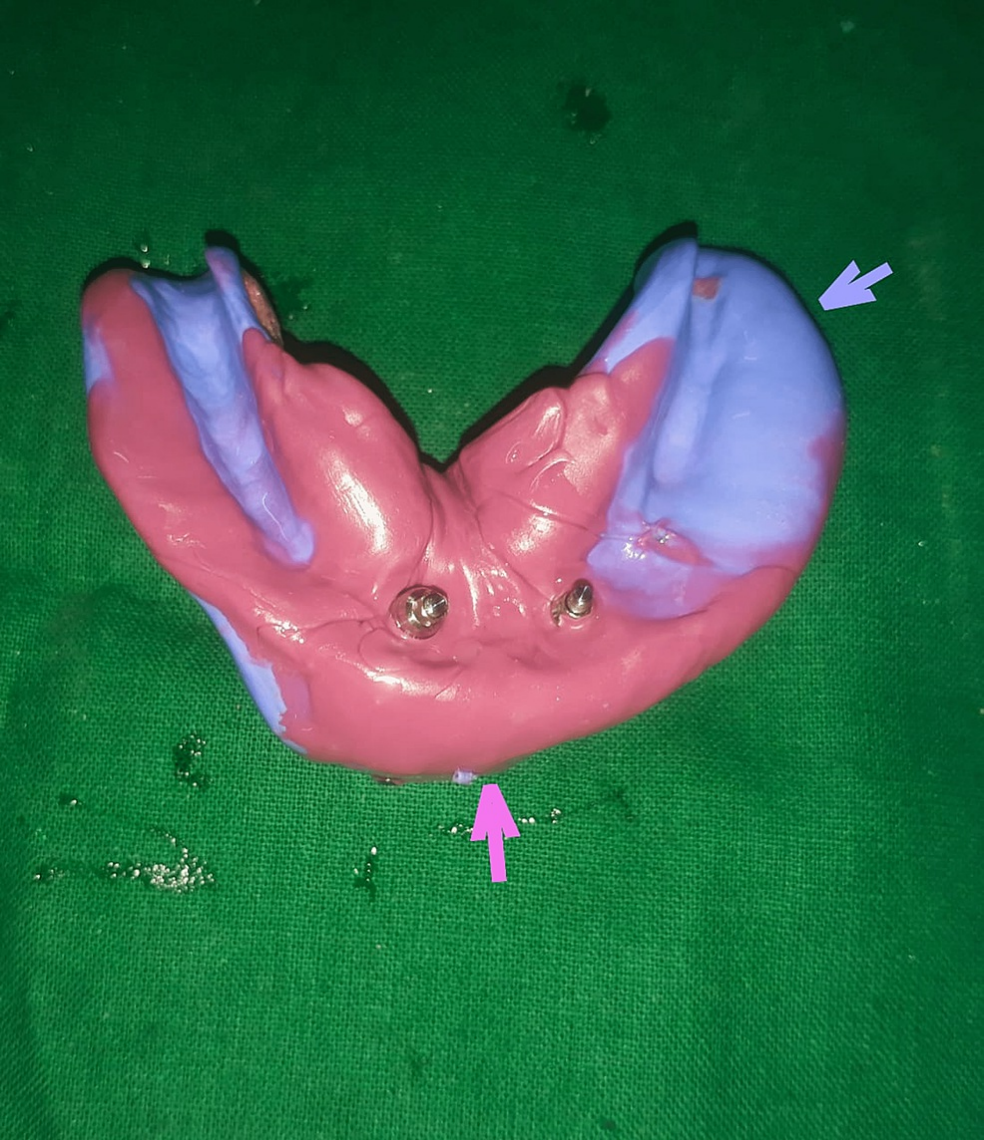
Open tray implant impression with Asilicone soft putty (blue arrow) and monophase (pink arrow).

Mouth opening was also slightly improved by the use of jaw exercises. Open tray impressions in Monophase and Putty (Aquasil Monophase, Dentsply, Aquasil soft putty, Dentsply, USA) were made using a special split tray (DPI, India) (Figure 5).

**Figure 6:**
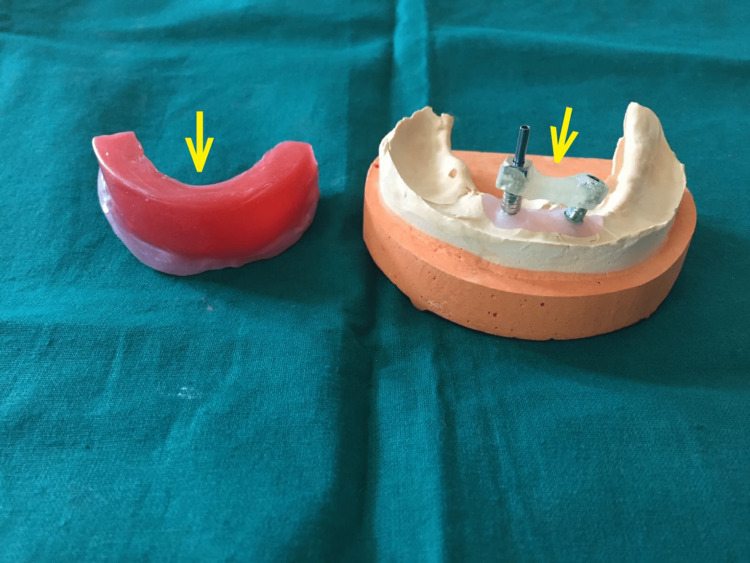
Mandibular Occlusal rim (arrow) and verification jig (arrow).

Healing abutments were placed back over mesial implants. Later, Jig verification was carried out and was satisfactory (Figure 6).

**Figure 7:**
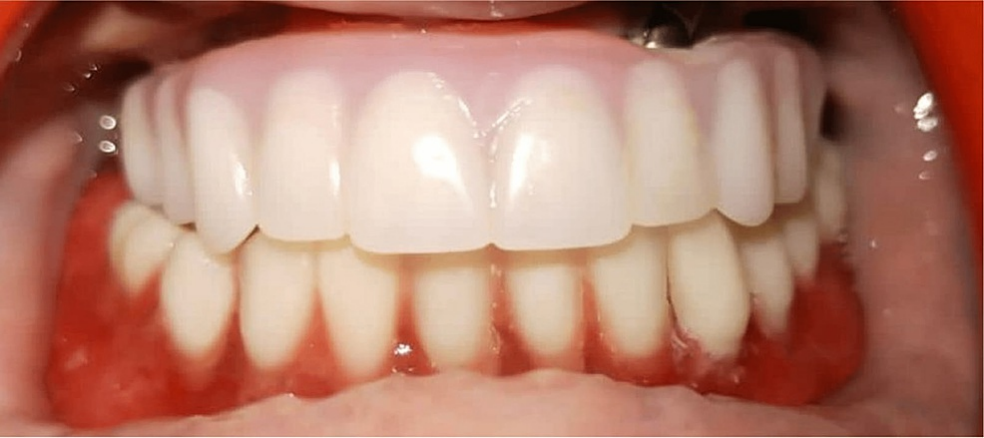
Try in verification with mandibular acrylic teeth.

Jaw relation was completed with ball abutments (H2mm, Dia 2.5mm, Ti, Norris Medical, Israel) screwed on 42 and 32 implants. Two days later, a try-in verification using trial dentures was performed (Figure 7).

**Figure 8:**
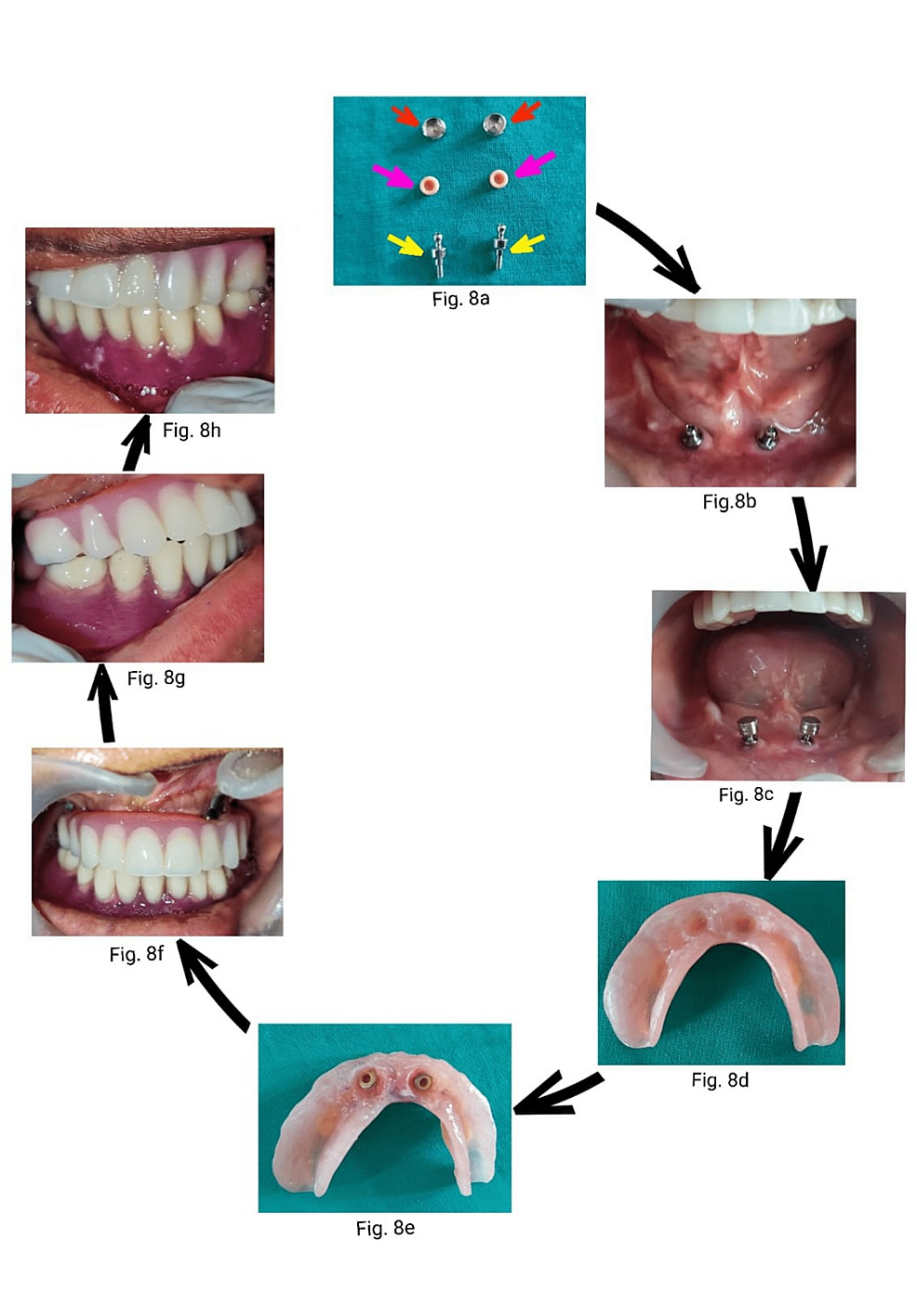
Flow chart showing sequences: ball abutments (yellow arrow), nylon caps (pink arrow) and metal encapsulators (red arrow) (8a), ball abutments intraoral view (8b), metal encapsulator over ball abutments (8c), denture intaglio surface prepared for nylon inserts (8d), nylon caps placed (8e), frontal view of mandibular overdenture (8f), right lateral view (8g), left lateral view (8h).

The final processed overdenture was inserted over ball abutments using a chairside relining technique using a metal encapsulator and the associated soft nylon cap (Norris Medical, Israel) (Figure 8a-8h).

**Figure 9:**
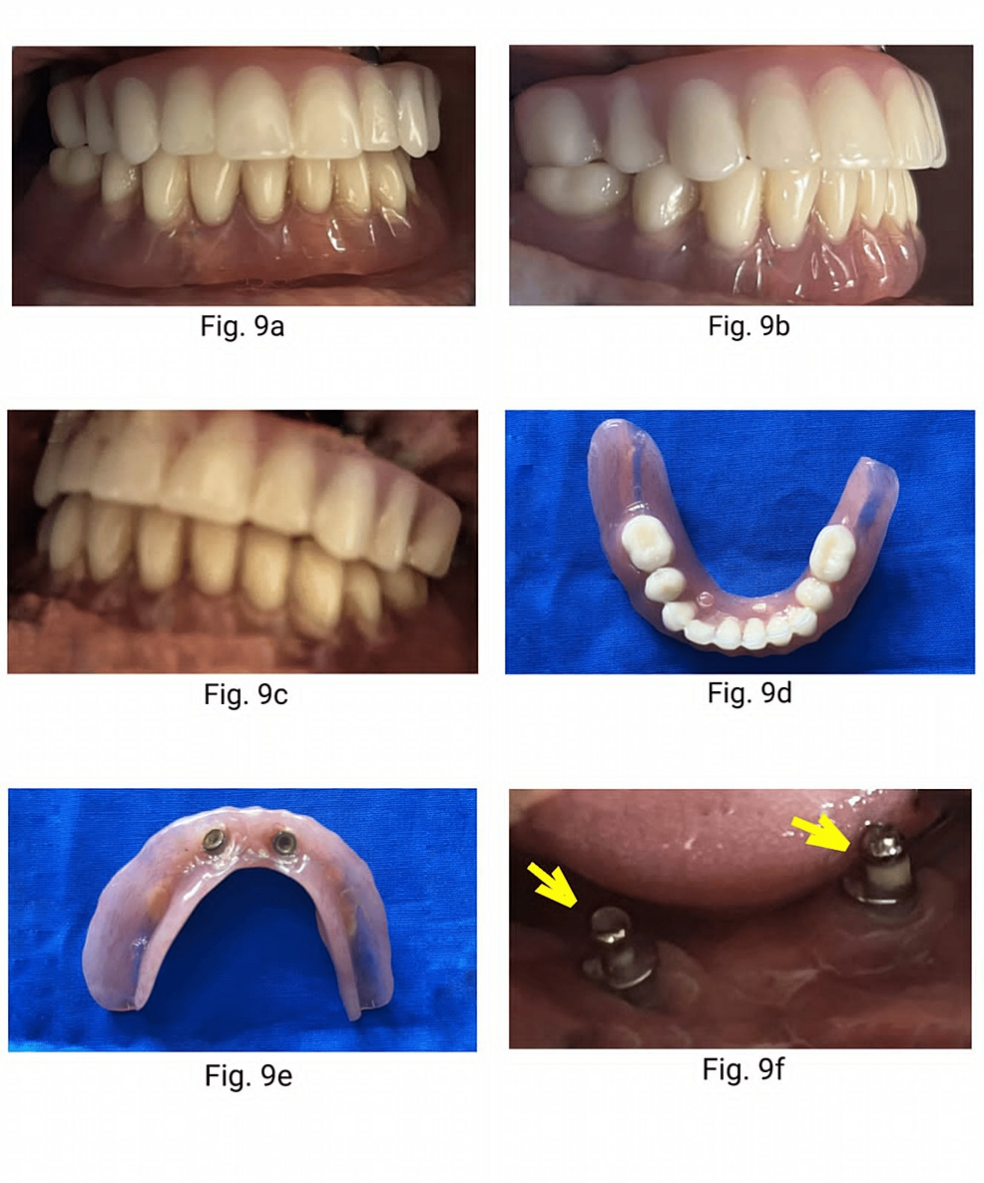
One-year follow-up: Frontal view of mandibular overdenture (9a), right lateral view (9b), left lateral view (9c), occlusal view (9d), intaglio view (9e), oral view of ball abutments (9f) (arrows).

**Figure 10:**
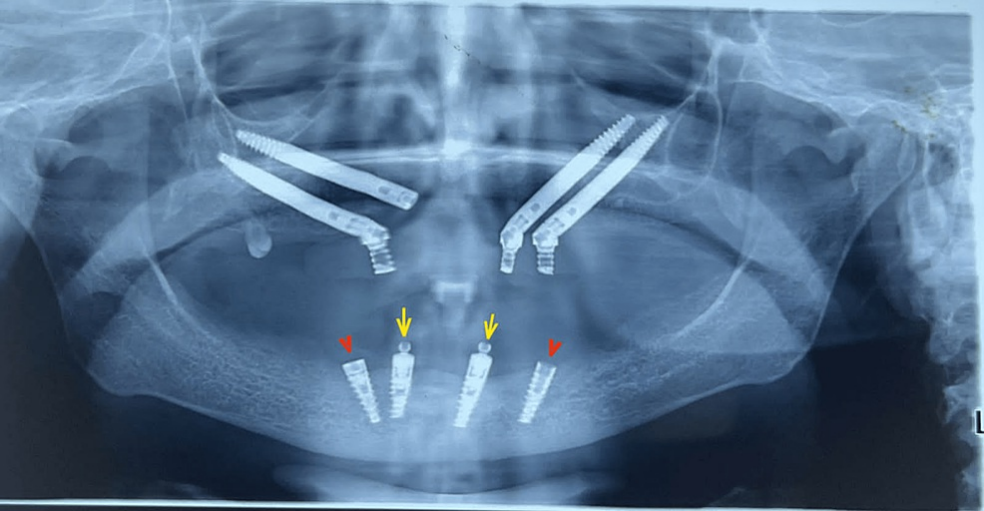
One-year follow-up: Orthopantogram showing two mesial functional implants (yellow arrows) and two distal dummy implants (red arrowheads).

The patient was educated on post-denture instructions with particular emphasis on the importance of maintenance of good oral hygiene. Follow-up was done three, six, and 12 months after denture insertion. Review at 12 months of completion was very encouraging (Figure 9a-9f) (Figure 10).

## Discussion

Judicious patient selection is ideal for positive dental implant treatment outcomes. Indications for such a treatment are based on multiple local and systemic factors influencing bone and soft tissue. These factors should be considered before proceeding to any dental implant therapy to predict the success of the overall treatment [13].

Prosthodontic rehabilitation with implants has successfully treated unique care patients, including individuals with Down syndrome [14]. Oral hygiene conditions and the level of intellectual disabilities should be clinically assessed. The repeated motivation of oral hygiene instructions for individuals with Down syndrome is also a critical factor in the treatment outcome [15]. Trias et al. concluded that more dental anomalies were identified in Down syndrome patients than in non-Down syndrome patients. Therefore periodic dental and orthodontic checkups were essential to maintain good oral hygiene [16]. 

The sudden unexpected change in the treatment plan initiated from the patient side was a total surprise. Even after several leading questions, there was no exact answer given. It had been presumed that this could be due to a change in the Patient's behavioral pattern.

Alqahtani et al. [17] presented the treatment of the Patient with Down syndrome with intellectual disability diagnosed with congenital and acquired tooth loss with clinical occlusal discrepancies. The absence of dental prostheses can impair patients' quality of life who require such restorations. Implant-supported dentures and bar construction improve the retention of prostheses in atrophied jaws [18]. Ribeiro et al. [19] reported implant-supported total prosthetic restoration with a hybrid prosthesis which the Patient accepted well. Satir et al. [20] reported mandibular canal variations in the Turkish population. This Patient exhibited normal canal morphology.

At one year follow-up, the Patient exhibited good oral hygiene, except for a small amount of plaque accumulation around ball abutments. Overdenture hygiene was surprisingly good. 

## Conclusions

Dental implant therapy, especially implant-supported overdenture, proves to be a viable treatment option in patients with Down syndrome. A one-year follow-up of this patient was highly successful and very much encouraging. Good oral hygiene and long-term maintenance are important for overall success. Change of treatment plan by the patient (primarily due to mood swings) from implant fixed hybrid to removable implant prosthesis still proved beneficial for the patient.
